# Psychological Distress, Fatigue, and Post‐Traumatic Stress Disorder Among Medical Staff Facing the Acute Watery Diarrhea Outbreak in Syria, Difficulties and Solutions: A Mixed‐Methods Study

**DOI:** 10.1002/hsr2.71958

**Published:** 2026-04-02

**Authors:** Ahmad Yamen Arnaout, Abdallah Aladna, Hassan Alshaker, Muhammad Besher Shabouk, Ibrahim Arnaout, Omar Najjar

**Affiliations:** ^1^ Internal Medicine, Aleppo University Hospital University of Aleppo Aleppo Syrian Arab Republic; ^2^ Faculty of Medicine University of Aleppo Aleppo Syrian Arab Republic

**Keywords:** cholera outbreak, post traumatic stress disorder, psychological distress

## Abstract

**Background:**

Cholera continues to be a significant public health threat, particularly in underserved regions with overcrowding and poor sanitation. The 2022–2023 cholera outbreak in Syria, particularly in Aleppo, overwhelmed medical staff, who faced extreme stress, extended work hours, and department closures. This cross‐sectional study examines the association between this outbreak and the psychological distress, fatigue, and post‐traumatic stress disorder (PTSD) experienced by healthcare workers.

**Methods:**

This cross‐sectional study, conducted at Aleppo University Hospital between September 20 and October 20, 2022, adhered to STROBE guidelines. A total of 75 healthcare providers voluntarily participated by completing a structured questionnaire that assessed socio‐demographic data and utilized standardized instruments to measure psychological distress, fatigue, and PTSD symptoms. In addition, qualitative data were collected to explore the challenges faced by healthcare workers during the outbreak and to gather their suggestions for better management in the future.

**Results:**

The findings indicate that most healthcare providers experienced low to moderate psychological distress and fatigue during the cholera outbreak, with moderate psychological distress being the most prevalent (84%, *n* = 63), followed by moderate fatigue in almost two‐thirds of the participants (62.7%, *n* = 47). Conversely, PTSD symptoms were rare, with only 8% (*n* = 6) of participants indicating PTSD according to the PCL‐5 questionnaire. Notably, the majority of medical staff perceived the cholera outbreak as less severe or more manageable compared to the COVID‐19 pandemic. However, a smaller number acknowledged that learning from the previous pandemic conditions mitigated the impact of the cholera outbreak. In terms of challenges faced, inadequate hospital resources and medical staffing were frequently cited, alongside gaps in patient awareness regarding the seriousness of the disease. Suggested solutions emphasized the need for better resource allocation, focused medical staff training, and enhanced hygiene awareness campaigns.

**Conclusion:**

The cholera outbreak in Syria has exerted significant psychological and physical strain on medical staff, underscoring the urgent need for targeted interventions to support their mental health and well‐being. Addressing these challenges is vital for sustaining an effective healthcare response during current and future public health crises.

**Abbreviations**
CFQ: Chalder Fatigue Scale
PSS: Perceived Stress Scale
PTSD: post‐traumatic stress disorder
STROBE: Strengthening the Reporting of Observational studies in Epidemiology
WHO: World Health Organisation

## 1 Introduction

Acute watery diarrhea (AWD) refers to the sudden onset of watery stools and is a common symptom of various gastrointestinal infections. According to the World Health Organization, when AWD is specifically caused by the bacterium Vibrio cholerae, it is classified as cholera. This distinction is important because while all cholera cases are a form of AWD, not all AWD cases are cholera [1].

Cholera, a longstanding infectious disease, remains a significant public health concern in many impoverished regions globally. Factors such as overcrowding, poverty, and inadequate water and sanitation infrastructure contribute to the increased vulnerability to cholera outbreaks [2, 3]. The epidemiology of cholera continues to evolve in various regions across Asia, Africa, and the Americas, where the disease is endemic [2, 3].

Annually, scientists estimate there are between 1.3 and 4.0 million cases of cholera worldwide, resulting in 21,000 to 143,000 deaths attributed to the disease [4]. On September 10, 2022, Syria officially declared a cholera outbreak. By September 30, surveillance data had reported a total of 10,039 suspected cases across 13 out of the 14 governorates in Syria [5]. From August 25, 2022, to February 15, 2023, approximately 92,649 suspected cases were documented across all governorates, with Aleppo alone recording 22,123 cases. During this interval, a total of 101 cholera‐related deaths were documented, with the majority (46 deaths) occurring in Aleppo [6].

The Syrian healthcare system was already in a state of profound crisis following over a decade of war, which had devastated infrastructure, depleted medical supplies, and led to a critical shortage of healthcare professionals. This pre‐existing strain significantly weakened the system's capacity to respond to a new public health emergency.

The healthcare profession involves making critical decisions under significant stress. Emergency physicians, in particular, are highly susceptible to burnout, with 35%–77.8% acknowledging substantial risks, as reported by Boutou (2019). Factors such as heavy workload, violence, exposure to traumatic events, unmanaged stress, conflicts between work and family responsibilities, and inadequate staffing have been identified in emergency medicine [7, 8].

With the onset of the cholera outbreak in Aleppo, healthcare personnel faced a surge in critically ill patients, leading to extended work hours and the temporary closure of hospital departments to concentrate efforts on cholera cases. For example, the influx of thousands of cholera patients exacerbated the already dire shortage of IV fluids, antibiotics, and clean water, pushing the healthcare workforce to its limits. This prolonged and demanding situation imposed considerable strain on the healthcare team, mirroring challenges documented during other medical crises. For instance, during the COVID‐19 pandemic, healthcare workers in Syria and globally reported severe fatigue and stress due to overwhelming patient loads and resource scarcity [9]. Similarly, studies on the Ebola and SARS outbreaks found high rates of psychological distress among medical staff, driven by the fear of infection, grueling workloads, and the emotional toll of caring for the critically ill [10].

In this high‐pressure environment, healthcare workers experienced elevated levels of stress, anxiety, and burnout, heightening their susceptibility to psychological conditions. This cross‐sectional study aims to investigate the association between the cholera outbreak and the prevalence of post‐traumatic stress disorder (PTSD), stress levels, and fatigue among healthcare workers.

## 2 Methods

### 2.1 Study Design and Participant's Involvement

This mixed‐methods, cross‐sectional study was conducted at Aleppo University Hospital in Aleppo, Syrian Arab Republic, following the Strengthening the Reporting of Observational Studies in Epidemiology (STROBE) guidelines for cross‐sectional studies [11]. Participants were recruited from September 20 to October 20, 2022, including adult medical staff at the hospital who voluntarily agreed to participate in the survey. Given the operational constraints of the active outbreak and security situation, a consecutive sampling method was employed. All eligible HCWs (defined as adult medical doctors, nurses, and clinical support staff directly involved in patient care during the outbreak) present on shift during the study period were invited to participate. Participation was voluntary. The final sample of 75 represents a feasibility sample based on accessibility and willingness to participate during the crisis period. Data collection was conducted using a validated, structured questionnaire, with the initial section collecting socio‐demographic information such as age and gender.

### 2.2 Variables and Measurement

#### 2.2.1 Psychological Distress

Daily stress levels were assessed using the Perceived Stress Scale (PSS), which is a widely‐used measure for evaluating global perceived stress and its impact on health and disease. The PSS total score was calculated based on participants' responses to a series of questions related to monthly stress levels and overall health status. The scale comprises 10 items, each rated from 0 to 4, yielding a possible total score between 0 and 40. Based on the total score, perceived stress levels were categorized as low (PSS 0–13), moderate (PSS 14–26), or high perceived stress (PSS 27–40). Moderate stress may indicate a manageable level that could impact daily functioning, while severe stress may reflect significant impairment, potentially leading to adverse long‐term health effects. This differentiation allows for targeted interventions that cater to the specific needs of individuals experiencing varied levels of stress [12].

#### 2.2.2 Fatigue Measurement

Fatigue levels were assessed using the Chalder Fatigue Scale (CFQ), which is specifically designed to measure the extent and severity of fatigue in both clinical and non‐clinical epidemiological studies. The CFQ consists of 11 items, where fatigue is evaluated across a spectrum, with responses ranging from “not at all” to “very much.” Interpretation of fatigue levels is categorized based on total scores: scores from 0 to 11 indicate no fatigue, while scores from 12 to 33 reflect increasing levels of fatigue severity. By providing a more nuanced understanding of fatigue levels, this classification aids in recognizing specific patterns in fatigue that may correlate with stress or other health‐related factors [13].

#### 2.2.3 Post‐Traumatic Stress Disorder (PTSD)

To evaluate PTSD among participants, the PCL‐5 questionnaire was utilized. This self‐report tool assesses the presence of PTSD symptoms by requiring individuals to reflect on their experiences since a traumatic event. Scores were analyzed to estimate the likelihood of PTSD, with specific criteria outlined for diagnosis: individuals must moderately endorse symptoms across all four clusters—intrusion, avoidance, negative alterations in cognition and mood, and alterations in arousal and reactivity. A minimum score of 33 on the PCL‐5 suggests a probable diagnosis of PTSD [14]. In addition to the standardized scales, open‐ended questions were used to qualitatively explore participants' self‐reported difficulties faced during the outbreak and their suggestions for potential solutions or support mechanisms. This qualitative data were collected to provide context and depth to the quantitative findings. A thematic analysis of these responses is planned for a separate publication.

#### 2.2.4 Other Variables

Participants were categorized according to the World Health Organization's Smoking and Tobacco Use Policy, distinguishing between daytime smokers who consume any tobacco product daily and occasional smokers who smoke, but not on a daily basis [15].

### 2.3 Statistical Methods

The data were analyzed using SPSS PC version 24.0 statistical software. Descriptive statistics, including mean, standard deviation, frequencies, and percentages, were used to describe both quantitative and categorical variables. All results of the statistical inference tests were interpreted at a 95% confidence level, using a significance level of 0.05 with two‐tailed hypothesis. There was no missing data. The normal distribution suitability of numerical variables was tested using the Shapiro‐Wilk test [16].

Responses to open‐ended questions were analyzed using inductive thematic analysis. The authors independently reviewed responses to identify recurring codes, which were then grouped into overarching themes (Challenges and Solutions). Discrepancies were resolved through discussion.

### 2.4 Ethics Approval and Consent to Participate

This research was conducted in accordance with the ethical guidelines outlined in the 1964 Declaration of Helsinki and its subsequent revisions, and with approval from the ethics committee at the Faculty of Medicine, University of Aleppo, Syria, with registered reference number 1932.

## 3 Results

### 3.1 Participants and Descriptive Data

A total of 75 healthcare providers who faced the cholera outbreak at Aleppo University Hospital were invited to take part in the survey, of which 49 (65.3%) were females and 26 (34.7%) were males. Participants could hold one or multiple roles, with the distribution as follows: 26.7% (*n* = 20) in management, 46.7% (*n* = 35) in observation and training, 90.7% (*n* = 68) as data collectors, and 4% (*n* = 3) in other roles.

The participants reported their average sleeping hours per night, as well as their smoking and stimulant consumption habits. The results showed that most of the participants slept between 6 and 8 h per night (62.7%, *n* = 47), followed by those who slept more than 8 h (21.3%, *n* = 16) and less than 6 h (16%, *n* = 12). Regarding smoking habits, only 4% (*n* = 3) of the participants were daily smokers, while 10.7% (*n* = 8) were occasional smokers, and 85.3% (*n* = 64) were non‐smokers. The most common number of stimulants consumed during the day was twice (38.7%, *n* = 29), followed by three times (22.7%, *n * = 17), once (18.7%, *n* = 14), more than three times (8%, *n* = 6), and rarely (12%, *n* = 9) (Table 1).

**Table 1:** Main characteristics of the participants.

		Total	Percent
Gender (male, %)		26	34.7%
Working positions during the outbreak (participants can choose more than one choice)			
	Observation and training	35	46.7%
	Management	20	26.7%
	Data collector for research	68	90.7%
	Other	3	4%
Smoking Status			
	Non‐smoker	64	85.3%
	An occasional smoker	8	10.7%
	A daily smoker	3	4%
Sleeping hours			
	Less than 6	12	16%
	Between 6–8	47	62.7%
	More than 8	16	21.3%
How much stimulants can be consumed during the day on a daily basis (coffee—tea)?			
	I only drink rarely	9	12%
	Once a day	14	18.7%
	Twice	29	38.7%
	Three times	17	22.7%
	More	6	8%

### 3.2 Psychological Distress, Fatigue, and Post‐Traumatic Stress Disorder Among Medical Staff

The participants exhibited a low prevalence of PTSD symptoms: with only 8% (*n* = 6) reporting PTSD, while 92% (*n* = 69) did not. The PSS scores showed that most of the participants had moderate stress scores (84%, *n* = 63), compared with 9.6% (*n* = 7) with high‐stress scores and 6.7% (*n* = 5) with low‐stress scores. Regarding fatigue, CFS scores indicated that more than half of the participants had moderate fatigue scores (62.7%, *n* = 47), while 34.7% (*n* = 26) had low fatigue scores and 2.7% (*n* = 2) had severe fatigue scores. The participants who had also dealt with the COVID‐19 pandemic (*n* = 46) were asked to compare it with the cholera outbreak. The majority of them (44%, *n* = 33) said that the cholera outbreak was better than the COVID‐19 pandemic, while 12% (*n* = 9) said that it was better because they learned from the COVID‐19 pandemic experience. Only 2.7% (*n* = 2) considered the cholera outbreak worse, and 2.7% (*n* = 2) felt it was similar to the COVID‐19 pandemic. The survey results suggest that the medical staff experienced low to moderate levels of psychological distress and fatigue due to the cholera outbreak. They also revealed that most of the medical staff regarded the cholera outbreak as less severe or more manageable than the COVID‐19 pandemic (Table 2).

**Table 2:** Outcomes of the study.

	Total	Percent
Post‐traumatic stress disorder		
Yes	6	8%
No	69	92%
The Chalder fatigue scale		
Low fatigue (*n*, %)	26	34.7%
Moderate fatigue (*n*, %)	47	62.7%
Severe fatigue (*n*, %)	2	2.7%
Perceived Stress Scale		
Low stress (*n*, %)	5	6.7%
Moderate stress (*n*, %)	63	84%
High stress (*n*, %)	7	9.6%
How do you compare this outbreak with COVID‐19 Pandemic?		
Better	33	44%
Better because we learned from COVID‐19 pandemic	9	12%
Same	2	2.7%
Worse	2	2.7%

### 3.3 Difficulties and Solutions

Difficulties as reported by participants were:

- The hospital was overwhelmed with a surge of patients arriving simultaneously, yet had only a limited number of available beds to accommodate them.
- The hospital has reached its capacity and is lacking medical staff and accurate diagnostic tests. Additionally, there is a shortage of necessary tools and devices for certain measurements, such as blood pressure and oximeter devices.
- Resident doctors also lack understanding of the importance and goals of scientific research or the research projects they are involved in.
- Some members of society are not aware of the seriousness of the situation, making it difficult to communicate with patients during follow‐up. This has led to some patients not describing their symptoms accurately and taking medications and antibiotics without consulting a doctor.
- Furthermore, there is a shortage of laboratory kits and poor in cooperation and communication between clinical and research teams.

Some solutions as reported by participants were:

- Enhance training and education for medical staff: Provide specialized training and education for medical staff on how to diagnose, treat, and prevent cholera. This can be achieved through partnerships with international organizations or by inviting experienced medical professionals to train local staff.
- Establish cholera treatment centers: Set up dedicated treatment centers specifically for cholera patients to ensure they receive the appropriate care. These centers could also serve as hubs for training and education for medical staff.
- Distribute clean water and sanitation supplies: Since cholera is often spread through contaminated water, providing clean water and sanitation supplies to affected areas is crucial in preventing further transmission of the disease.
- Conduct public health awareness campaigns: Educate the public about cholera symptoms, transmission methods, and preventive measures. This could include distributing informational materials, hosting community workshops, and working with local leaders to spread awareness.
- Coordinate with international aid organizations: Partner with international aid organizations to access resources, expertise, and funding to help confront the outbreak. These organizations may have experience dealing with cholera outbreaks in other parts of the world and can provide valuable support.
- Implement vaccination campaigns: If available, administer cholera vaccines to high‐risk populations to help prevent further spread of the disease.
- Monitor and track cases: Establish a system for monitoring and tracking cholera cases to better understand the scope of the outbreak and target resources where they are most needed.
- Collaborate with neighboring countries: Work with neighboring countries that may have experience in managing cholera outbreaks or possess resources that can be shared to confront the outbreak in Syria.

## 4 Discussion

The cholera outbreak in Syria between 2022 and 2023 hit 13 out of its 14 governorates inflicting major damage to the already‐compromised economic and healthcare sectors [2, 5]. The outbreak was at its peak in Aleppo Governorate, which alone accounted for a quarter of Syria's registered cases and nearly half of the death toll [2]. Our article aimed to investigate the psychological burden of this outbreak on frontline healthcare workers at Aleppo University Hospital, the largest still‐operating hospital in Aleppo after years of war. The primary focus was to assess psychological fatigue as well as detect PTS symptoms in our medical staff.

Our results have shown that although most of our population did not experience PTS symptoms (8%), while most of them had at least moderate (84%) or severe (9.6%) psychological stress. Although this is the first study to examine the psychological impacts of a cholera outbreak, similar outcomes have been observed in other infectious disease outbreaks. For instance, a study by Kisely et al. (2020) [17, 18] during the COVID‐19 pandemic found that healthcare workers experienced high levels of anxiety, depression, and PTSD, with prevalence rates for PTSD ranging from 10% to 20% in some studies. Similarly, research during the Ebola outbreak in Sierra Leone reported substantial mental health impacts, including PTSD, among healthcare providers due to the extreme stress and traumatic experiences encountered in managing the epidemic [19].

When asked to compare the psychological impact of the recent cholera outbreak to the COVID‐19 pandemic, the majority of the studied population (44%) stated that the cholera outbreak had a more manageable effect. This suggests that prior exposure to pandemic conditions might enhance preparedness and resilience among medical staff. Similar observations were made in a study by Lai et al. (2020) [20], which found that healthcare workers who had previous experience with infectious disease outbreaks, such as SARS or H1N1, reported better coping mechanisms and lower levels of psychological distress during the COVID‐19 pandemic. However, it is crucial to note that the perceived manageability did not eliminate the significant stress and fatigue experienced during the cholera outbreak. The difference in perceived severity could also be related to the different natures of the diseases and their respective contexts. For example, cholera patients are usually less prone to death and can be more easily managed than COVID‐19 patients. Also, the risk of contracting the disease through patient contact in cholera is extremely less likely and less life‐threatening than that of COVID‐19. Subsequently, the precautions needed in cholera were less strict and therefore less mentally stressful. Finally, the cholera outbreak was local rather than global. Hence, the stress that usually results from the media was less prominent.

### 4.1 Challenges and Solutions: Qualitative Insights

Participants identified systemic challenges rooted in Syria's protracted crisis, including inadequate hospital capacity, staffing shortages, and limited diagnostic tools. Proposed solutions—enhanced training, dedicated cholera centers, and public awareness campaigns—align with interventions from other outbreaks. For example, during Ebola, international collaboration and training programs mitigated resource gaps. These insights were derived from open‐ended survey responses, reflecting recurring themes rather than consensus. While not exhaustive, they highlight critical areas for intervention.

Similar difficulties and recommended interventions have been reported in other settings. For instance, during the Ebola outbreak, healthcare workers faced significant challenges due to resource shortages and inadequate training, which were mitigated through international collaboration and enhanced training programs [21]. The importance of adequate resources and mental health support for healthcare workers during infectious disease outbreaks has also been emphasized in numerous studies, highlighting the need for comprehensive strategies to support medical staff [22].

### 4.2 Clinical Implications

The findings underscore the need for targeted interventions to support the mental health of healthcare workers during infectious disease outbreaks. Potential strategies include:

- **Enhanced Training and Education**: Providing healthcare workers with specialized training to manage infectious disease outbreaks can improve their competence and confidence, thereby reducing stress and fatigue. This approach was effective in previous outbreaks, where training programs significantly improved healthcare workers' preparedness and psychological resilience [23].
- **Mental Health Support Services**: Establishing mental health support services, including counseling and stress management programs, can help mitigate the psychological impact of outbreaks on medical staff. Mental health support was found to be crucial during the SARS outbreak, where the availability of counseling services significantly reduced psychological distress among healthcare workers [24, 25].
- **Resource Allocation**: Providing adequate resources, including medical supplies and diagnostic tools, can reduce the operational burden on healthcare workers, thereby alleviating stress and fatigue. Adequate resource allocation was a key factor in reducing stress and improving the overall mental health of healthcare workers during the H1N1 outbreak [26].

### 4.3 Limitations of the Study

This study has several limitations. The cross‐sectional design restricts the ability to determine causality. The sample size is relatively small and may not accurately reflect the experiences of all healthcare workers in Aleppo or other regions affected by the cholera outbreak. It is crucial to highlight unique local factors, particularly the prolonged conflict that has significantly strained hospital infrastructure. The ongoing instability has led to resource scarcity and increased stress on healthcare workers, which may skew our outcomes in ways that are not evident in other contexts. Additionally, self‐reported measures may introduce response biases. Future studies should consider longitudinal designs and larger, more diverse samples to better understand the long‐term psychological impacts of infectious disease outbreaks on healthcare workers.

## 5 Conclusion

The study highlights the psychological distress, fatigue, and PTSD among healthcare workers during the cholera outbreak in Aleppo. Despite the manageable nature of the cholera outbreak in comparison to the COVID‐19 pandemic, the findings underscore the urgent need for interventions to support the mental health and well‐being of medical staff during such crises. Implementing targeted strategies, including enhanced training, mental health support services, and adequate resource allocation, is essential to mitigate the psychological impact on healthcare workers.

## Author Contributions

**Ahmad Yamen Arnaout:** conceptualization, methodology, data curation, formal analysis, validation, writing – original draft, writing – review and editing. **Abdallah Aladna**, **Hassan Alshaker**, and **Muhammad Besher Shabouk:** investigation, writing – original draft preparation, writing – reviewing and editing. **Ibrahim Arnaout:** investigation, data curation, writing – original draft preparation, writing – reviewing and editing. **Omar Najjar:** writing – original draft preparation, writing – reviewing and editing.

## Funding

The authors received no specific funding for this work.

## Consent

The authors have nothing to report.

## Conflicts of Interest

The authors declare no conflicts of interest.

## Transparency Statement

The lead author Ahmad Yamen Arnaout affirms that this manuscript is an honest, accurate, and transparent account of the study being reported; that no important aspects of the study have been omitted; and that any discrepancies from the study as planned (and, if relevant, registered) have been explained.

## Data Availability

The data generated and analyzed during this study are available from the corresponding author upon reasonable request.
